# Cortactin Is Involved in the Entry of *Coxiella burnetii* into Non-Phagocytic Cells

**DOI:** 10.1371/journal.pone.0039348

**Published:** 2012-06-22

**Authors:** Eliana M. Rosales, Milton O. Aguilera, Romina P. Salinas, Sergio A. Carminati, María I. Colombo, Narcisa Martinez-Quiles, Walter Berón

**Affiliations:** 1 Instituto de Histología y Embriología, Facultad de Ciencias Médicas, Universidad Nacional de Cuyo - CONICET, Mendoza, Argentina; 2 Microbiología II, Facultad de Farmacia, Universidad Complutense de Madrid, Madrid, España; University of São Paulo, Brazil

## Abstract

**Background:**

Cortactin is a key regulator of the actin cytoskeleton and is involved in pathogen-host cell interactions. Numerous pathogens exploit the phagocytic process and actin cytoskeleton to infect host cells. *Coxiella burnetii*, the etiologic agent of Q fever, is internalized by host cells through a molecular mechanism that is poorly understood.

**Methodology/Principal Finding:**

Here we analyzed the role of different cortactin motifs in the internalization of *C. burnetii* by non-phagocytic cells. *C. burnetii* internalization into HeLa cells was significantly reduced when the cells expressed GFP-cortactin W525K, which carries a mutation in the SH3 domain that renders the protein unable to bind targets such as N-WASP. However, internalization was unaffected when the cells expressed the W22A mutant, which has a mutation in the N-terminal acidic region that destroys the protein’s ability to bind and activate Arp2/3. We also determined whether the phosphorylation status of cortactin is important for internalization. Expression of GFP-cortactin 3F, which lacks phosphorylatable tyrosines, significantly increased internalization of *C. burnetii*, while expression of GFP-cortactin 3D, a phosphotyrosine mimic, did not affect it. In contrast, expression of GFP-cortactin 2A, which lacks phosphorylatable serines, inhibited *C. burnetii* internalization, while expression of GFP-cortactin SD, a phosphoserine mimic, did not affect it. Interestingly, inhibitors of Src kinase and the MEK-ERK kinase pathway blocked internalization. In fact, both kinases reached maximal activity at 15 min of *C. burnetii* infection, after which activity decreased to basal levels. Despite the decrease in kinase activity, cortactin phosphorylation at Tyr421 reached a peak at 1 h of infection.

**Conclusions/Significance:**

Our results suggest that the SH3 domain of cortactin is implicated in *C. burnetii* entry into HeLa cells. Furthermore, cortactin phosphorylation at serine and dephosphorylation at tyrosine favor *C. burnetii* internalization. We present evidence that ERK and Src kinases play a role early in infection by this pathogen.

## Introduction

Phagocytosis is the process that cells have developed for the engulfment of particulate material such as apoptotic cells, cell debris and, also, inert particles. Furthermore, phagocytosis represents a crucial event that triggers host defense mechanisms against invading pathogens. Nevertheless, several pathogens have acquired different strategies to alter these mechanisms to survive and multiply within host cell, causing infectious diseases [1], [2]. The phagocytic process is initiated by a recognition step in which ligands on the particle surface bind receptors on the membrane of host cells [3]. The ligand-receptor interaction leads to actin cytoskeleton and membrane rearrangements that permit, first, particle engulfment and, later, particle sequestration into a phagosome which precedes phagosome maturation into a phagolysosome [4], [5].

Dynamic remodeling of the actin cytoskeleton is not only intimately involved in phagocytosis [6] but also in other essential cellular processes, including cell adhesion and motility [7], vesicle transport [8], [9], apoptosis [10] and endocytosis [11], all of which require dynamic remodeling of the actin cytoskeleton. There are numerous actin-associated proteins and several upstream signaling molecules that work in a coordinated way to control with exquisite precision the spatial and temporal assembly of actin structures, which can rapidly change in response to internal and external signals [12], [13]. Proteins of the Arp2/3 complex that function as nucleators of branched actin filaments are activated by interaction with members of the Wiskott-Aldrich syndrome protein (WASP) family and cortactin [14], [15]. Initial activation of WASP depends on its interaction with Rho family GTPases [9]. These multicomponent complexes of Arp2/3-WASP-cortactin are involved in cellular processes such as cell motility [16], endocytosis [17] and phagocytosis [18], [19]. Interestingly, some pathogens can regulate the host actin cytoskeleton during infection [20], [21].

Cortactin is a key regulator of the actin cytoskeleton, and it plays a crucial role in tumor cell invasion [22], ruffles and lamellipodium formation during integrin-mediated cell adhesion [23], [24] and podosome formation [25]. Cortactin is also an important component of the endocytic machinery [26]. It has emerged as a common target of pathogen-host cell interactions. For example, cortactin has been implicated in the adhesion of *Escherichia coli* [27] and in invasion by *Shigella*, *Neisseria*, *Chlamydia*, *Staphylococcus* and *Listeria*. The phosphorylation status of cortactin has been proposed to differentially regulate the invasion of many microbial pathogens. Cortactin is also involved in actin-based motility of many pathogens during their intracellular trafficking [28].

Cortactin possesses an N-terminal acidic domain (NTA) and F-actin-binding repeats that activate the Arp2/3 complex to initiate actin polymerization [29]. Cortactin also has a proline-serine-threonine-rich region (PST) that contains tyrosine residues critical for cortactin function. The C-terminal SH3 domain of cortactin binds various proteins, such as N-WASP proteins [30], [31]. The Verprolin Cofilin Acidic domain (VCA) of WASP members can also activate the Arp2/3 complex [32]. Theoretically N-WASP, cortactin and the Arp2/3 complex can form ternary complexes [32]. Cortactin is phosphorylated by tyrosine kinases (Src, Fer, Syk and Abl) and serine/threonine kinases (ERK and Pak) in response to a wide range of stimuli that induce cytoskeletal rearrangement, including growth factor stimulation, cell adhesion and hyperosmotic stress [33].

*Coxiella burnetii*, the causative agent of human Q fever, is an obligate intracellular bacterium found in a wide range of hosts, including livestock and humans. The primary route of infection in humans is inhalation of contaminated aerosols [34], [35]. Infected animals shed *C. burnetii* in their milk, urine and feces, and the bacteria are dispersed together with amniotic fluids and the placenta during birthing. These bacteria can survive for long periods in the environment, since they are highly resistant to heat, desiccation and common disinfectants.

*C. burnetii* inhabits mainly monocytes/macrophages but can infect a wide variety of cultured cell lines *in vitro* [36]. This bacterium resides in an acidic parasitophorous vacuole (PV) with late endosome-lysosome characteristics [37]–[39]. The PV also interacts with the autophagic pathway, acquiring autophagosomal features [37], [38], [40]. Interestingly, we have shown that PV biogenesis is regulated by actin and Rho family GTPases [41].

In this report we describe the involvement of cortactin in *C. burnetii* internalization into HeLa cells, a non-professional phagocyte cell line. We investigated the role of the Arp2/3-activating DDW motif in the N-terminal acidic region and of the SH3 domain at the C-terminus of cortactin during *C. burnetii* internalization. We observed that overexpression of cortactin mutated in the SH3 domain inhibits uptake of the bacterium, suggesting that the SH3 domain is important for internalization. We also analyzed the role of cortactin phosphorylation in internalization. By overexpressing cortactin mutants that are non-phosphorylatable and that mimic phosphorylation, we show that cortactin favors *C. burnetii* internalization in a tyrosine dephosphorylation- and/or serine phosphorylation-dependent manner. Furthermore, pharmacological inhibition of Src and ERK kinases reduce *C. burnetii* uptake. Our results indicate that phosphorylation status of cortactin affect internalization of *C. burnetii*.

**Figure 1:**
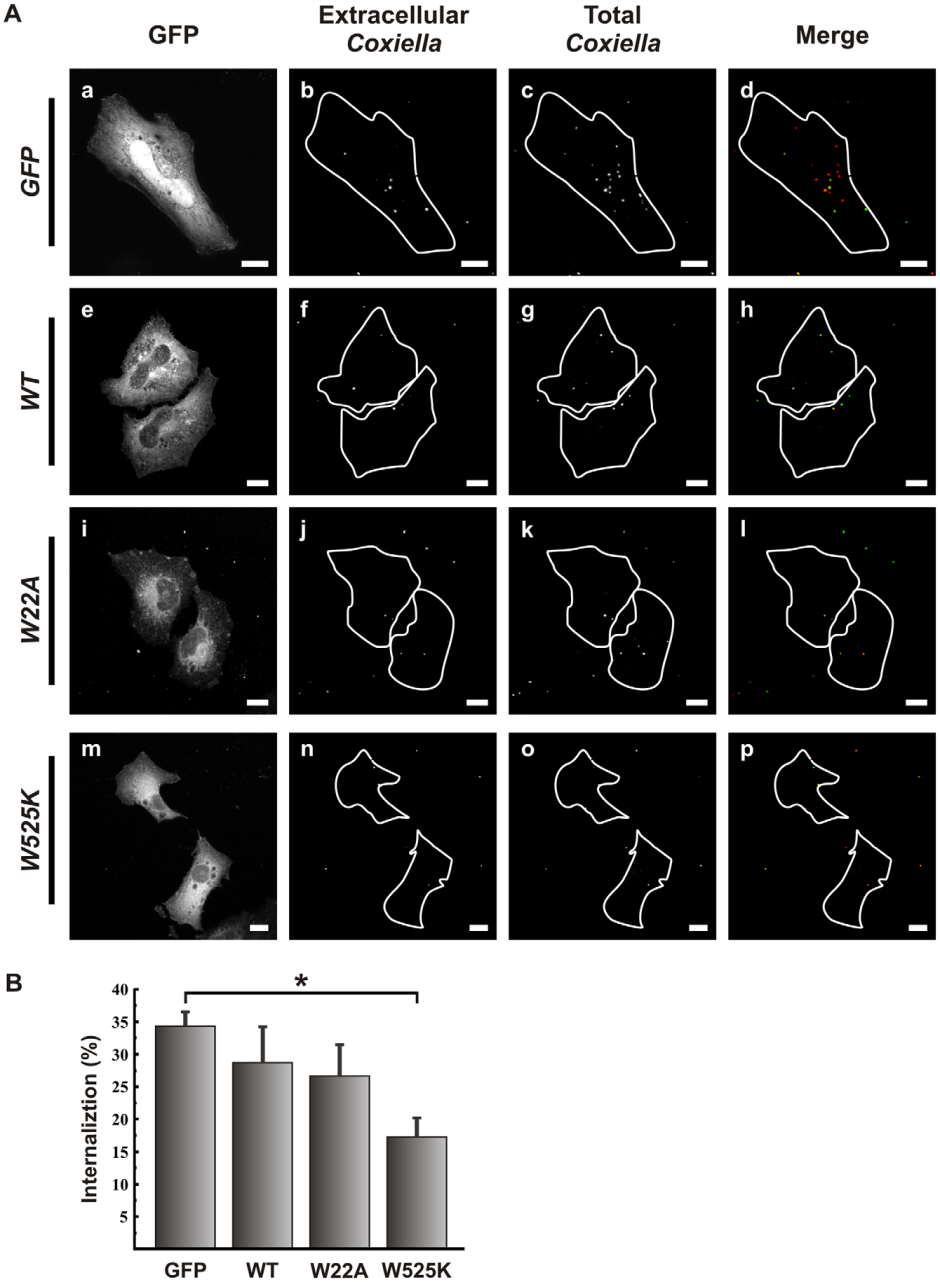
The SH3 domain of cortactin is important for *C. burnetii* internalization. (A) HeLa cells were transfected with pGFP-cortactin WT, GFP-cortactin W22A, a mutant that does not interact with Arp 2/3, or GFP-W525K, a mutant unable to bind and activate targets such as N-WASP. Transfected cells were infected for 4 h with *C. burnetii*. Cells were fixed and processed for immunofluorescence with a specific anti-*C. burnetii* antibody (see Methods). Cells were analyzed by confocal microscopy. In the merged images (panels d, h, l and p), extracellular *C. burnetii* are shown in green and red pseudocolors while intracellular *C. burnetii* are shown in red pseudocolor. Bars, 10 µm. (B) Quantification of *C. burnetii* internalized by transfected HeLa cells. Results are expressed as means ± SE of at least three independent experiments. *, P<0.05. (%), percentage of total number of bacteria.

## Methods

### Materials

Dulbecco’s Modified Eagle's Medium (D-MEM), fetal bovine serum (FBS), penicillin and streptomycin were obtained from Gibco BRL/Life Technologies (Buenos Aires, Argentina). Vectors encoding a fusion of green fluorescent protein (GFP) with cortactin WT (full-length cortactin) or GFP-cortactin 3F (cortactin mutated in the three tyrosine-phosphorylation sites recognized by Src) were kindly provided by S. Bourdoulous (Département de Biologie Cellulaire, Institut Cochin, Université Paris, Paris, France). Rabbit polyclonal anti-*Coxiella* antibody against Nine Mile phase II, clone 4 (RSA439) was generously provided by Dr. Robert Heinzen (Rocky Mountain Laboratories, NIAID, NIH, Hamilton, MT, USA). Secondary antibodies were purchased from Jackson ImmunoResearch Laboratories, Inc. (West Grove, PA, USA). Rabbit monoclonal anti-phosphocortactin (Tyr421) antibody was purchased from Abcam (MA, USA), mouse monoclonal anti-actin Ab-5 antibody was purchased from BD (Buenos Aires, Argentina), rabbit anti-phosphoSrc (Tyr416) (Cell Signaling Inc., MA, USA) and anti-Src antibodies were generously provided by Arlinet Kierbel (Montevideo Pasteur Institute, Montevideo, Uruguay), and mouse monoclonal anti-phosphoERK1/2 (Tyr204) and rabbit polyclonal anti-ERK antibodies were purchased from Santa Cruz Biotechnology (California, USA). The inhibitors PD98059 and SU6656 were from Invitrogen (Buenos Aires, Argentina) and CalBiochem (Darmstadt, Germany), respectively. Protease inhibitor cocktail was from Sigma (Buenos Aires, Argentina).

### Cell Culture

HeLa cells were grown in DMEM supplemented with 10% heat-inactivated FBS, 2.2 g/l sodium bicarbonate, 2 mM glutamine and 0.1% penicillin/streptomycin at 37°C under 5% CO_2_.

### Propagation of Phase II *Coxiella burnetii*

Clone 4 phase II Nine Mile strain of *C. burnetii* bacteria were provided by Ted Hackstadt (Rocky Mountain Laboratories, NIAID, NIH, Hamilton, MT, USA) and handled in a biosafety level II facility. Non-confluent Vero cells were cultured in T25 flasks at 37°C under 5% CO_2_ in DMEM medium supplemented with 5% FBS, 0.22 g/l sodium bicarbonate and 20 mM Hepes, pH 7 (MfbH). Cultures were infected with *C. burnetii* phase II suspensions for 6 days at 37°C under 5% CO_2_. After freezing at −70°C, the flasks were thawed, and the cells scraped and passed 20 times through a 27-gauge needle connected to a syringe. Cell lysates were centrifuged at 800×*g* for 10 min at 4°C. The supernatants were centrifuged at 24,000×*g* for 30 min at 4°C, and pellets containing *C. burnetii* were resuspended in phosphate-buffered saline (PBS; 10 mM sodium phosphate, 0.9% NaCl), aliquoted and frozen at −70°C.

### Infection of HeLa Cells with *Coxiella burnetii*

Cells (5×10^5^) were seeded on sterile glass coverslips placed in 24-well plates and grown overnight in MfbH medium. For infection, a 5-µl aliquot of *C. burnetii* suspension was added per well (Multiplicity of infection: ∼20). Cells were incubated for different lengths of time at 37°C under 5% CO_2_.

### Quantification of Internalized Bacteria by Indirect Immunofluorescence

To determine the number of internalized bacteria a double cycle antibody staining protocol was used [42]. Briefly, HeLa cells were fixed with 2% paraformaldehyde in PBS for 10 min at 37°C, washed with PBS and blocked with 50 mM NH_4_Cl in PBS. After washing, cells were incubated with rabbit antibody against *C. burnetii* (1∶1000) and donkey anti-rabbit secondary antibody conjugated with Cy3 (1∶600) in PBS containing 0.5% BSA (nonpermeabilizing conditions to label extracellular bacteria). After washing, cells were incubated with the same rabbit antibody against *C. burnetii* (1∶1000) and a donkey anti-rabbit secondary antibody conjugated with Cy5 (1∶600) in PBS containing 0.5% BSA and 0.05% saponin (permeabilizing conditions to label total bacteria: intracellular and extracellular bacteria). Coverslips were mounted with Mowiol and examined by confocal microscopy. The intracellular bacteria are expressed as a percentage of the total number of bacteria per cell.

### Cell Transfection

Cells were transfected for 6 h with 2 µg/ml pGFP empty vector or pGFP plasmids expressing fusions of GFP with wild-type cortactin (WT) or one of the following mutants: single point mutants, W22A and W525K; double mutants, S405/418D (SD) and S405/418A (2A); and triple mutants, Y421/466/482D (3D) and Y421/466/482F (3F). Cell transfection was carried out using Lipofectamine™ 2000 (Invitrogen, Buenos Aires, Argentina), according to the manufacturer’s instructions. After 6 h of transfection, the cells were washed and incubated for 18 h in MfbH medium at 37°C under 5% CO_2_.

### Western Blotting

Hela cells were cultured on 60-mm dishes and infected as described above for different lengths of time. After the indicated infection periods, cells were washed with PBS, scraped into ice-cold lysis buffer (50 mM Tris-HCl, pH 7.2, 1% Triton X-100, 0.5% deoxycholate, 0.1% SDS, 50 mM NaCl, 10 mM MgCl_2_, 2 mM Na_3_VO_4_, 10 mM NaF, 0.5 mg/ml DTT, 2 mM EDTA) supplemented with a protease inhibitor cocktail and kept on ice for 20 min. Lysates were clarified by centrifugation at 2000×*g* for 15 min at 4°C. Clarified lysates were transferred to clean tubes, mixed with Laemmli buffer and boiled for 5 min. The samples were resolved by SDS-PAGE and the proteins transferred to nitrocellulose membranes using standard procedures. Membranes were blocked for 2 h at 4°C in Tween-Tris-buffered saline (TTBS; 0.1% Tween 20, 100 mM Tris/HCl, 0.9% NaCl) supplemented with 5% BSA, then incubated overnight at 4°C with the appropriate primary antibodies. The membranes were washed three times with TTBS, then, incubated for 2 h at room temperature with appropriate peroxidase-conjugated secondary antibodies. Membranes were washed again with TTBS and developed using the ECL Western blotting system (GE Healthcare) according to the supplier’s recommendations. Blotting with anti-GAPDH or anti-actin antibody was carried out to provide loading controls. Band densitometry was carried out using ImageJ software (NIH, USA).

### Fluorescence Microscopy

HeLa cells were analyzed by confocal microscopy using an FV1000 Olympus Confocal Microscope and FV 10-ASW 1.7 Software (Olympus, Japan). Images were processed using ImageJ software.

### Statistical Analysis

Results were analyzed by the ANOVA test in conjunction with Tuckey and Dunnett tests.

**Figure 2:**
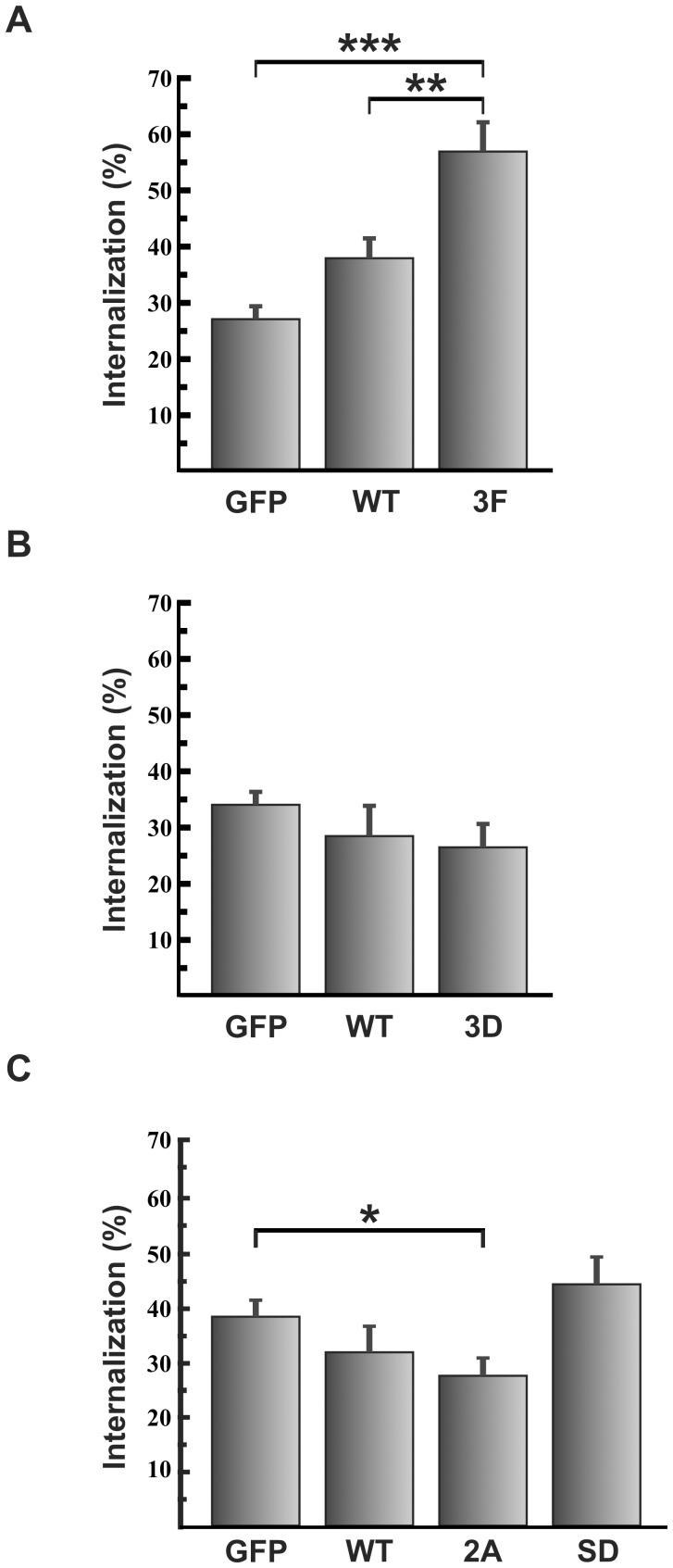
Cortactin mutants not phosphorylatable by Src and ERK stimulated and inhibited *C. burnetii* internalization, respectively. HeLa cells were transfected with pGFP, pGFP-cortactin WT (wild type) or plasmids encoding GFP fusions with one of the following cortactin mutants: (A) pGFP-cortactin 3F, a Src non-phosphorylatable mutant; (B) pGFP-cortactin 3D, which mimics cortactin phosphorylated by Src; or (C) pGFP-cortactin 2A, an ERK non-phosphorylatable mutant, or pGFP-cortactin SD, which mimics cortactin phosphorylated by ERK. Transfected cells were infected for 4 h with *C. burnetii*, fixed and processed for indirect immunofluorescence to determine intracellular *C. burnetii* (see Methods). Cells were analyzed by confocal microscopy. Results are expressed as means ± SE of at least three independent experiments. *, P<0.05; **, P<0.01; ***, P<0.001. (%), percentage of total number of bacteria.

**Figure 3:**
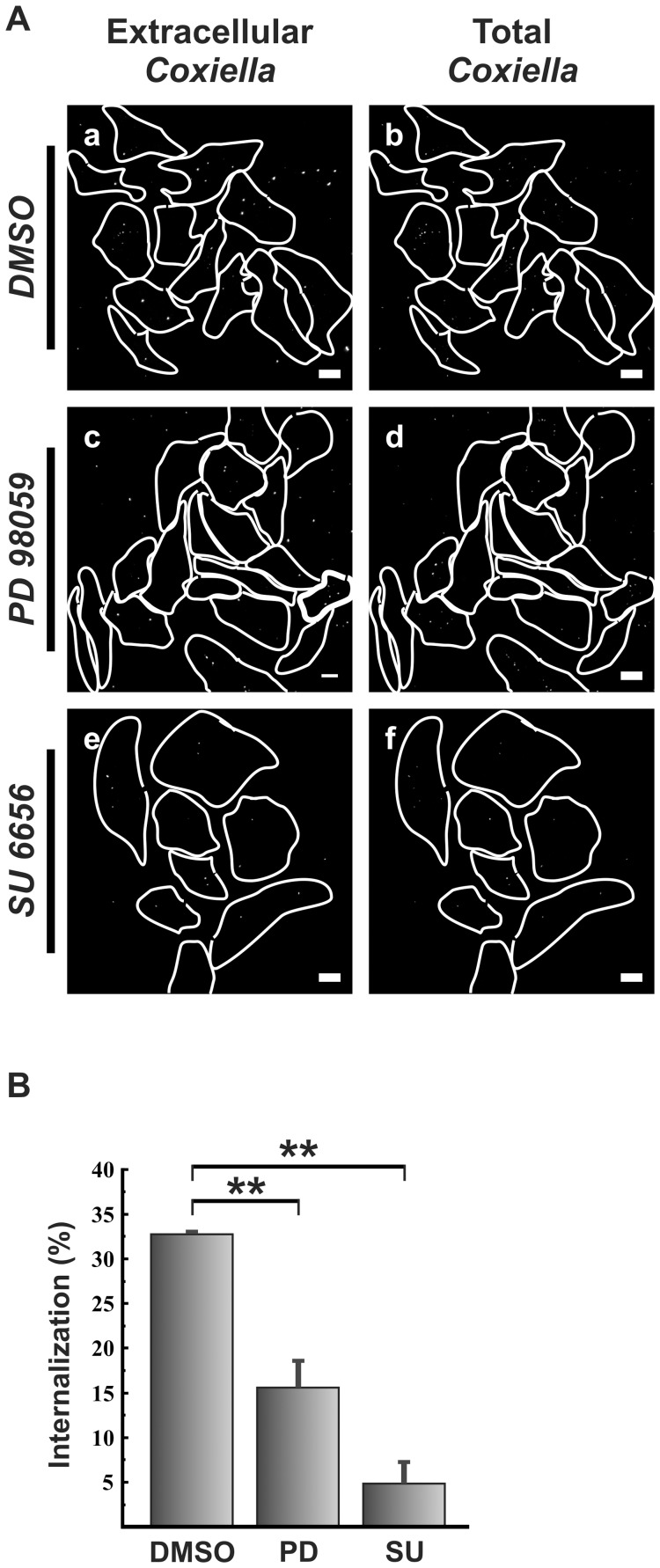
Src and ERK kinases are involved in *C. burnetii* internalization. (A) HeLa cells were incubated for 1 h at room temperature with 0.05% DMSO (control), 15 µM PD 98059 (MEK-ERK inhibitor) or 5 µM SU6656 (Src inhibitor). Then the cells were infected for 4 h with *C. burnetii* in the presence of the inhibitors. Cells were fixed and processed for indirect immunofluorescence using a specific anti-*C. burnetii* antibody (see Methods). Bars, 10 µm. (B) Quantification of *C. burnetii* internalized by treated HeLa cells. Results are expressed as means ± SE of three independent experiments. **, P<0.01. (%), percentage of the total number of bacteria.

## Results

### The SH3 Domain of Cortactin is Important for *C. burnetii* Internalization

Cortactin is an F-actin regulatory protein that plays an important function in various cellular processes such as cell adhesion, motility and endocytosis. However, its role in phagocytosis has been poorly characterized. Interestingly, cortactin is recruited to the contact sites made by several pathogens with the host plasma membrane during infection [28].

In its N-terminal acidic region (NTA), cortactin contains a short motif called DDW that binds and activates the Arp2/3 complex [29]. This motif is followed by 6.5 tandem repeats of a 37-residue sequence responsible for F-actin binding. It has a Src homology 3 (SH3) domain at the C-terminus that mediates the interaction with various proteins, including the Arp2/3-stimulating Wiscott-Aldrich protein N-WASP [43]. These interactions link actin remodeling to several specific processes. Mutations in cortactin that abrogate Arp2/3 activation (W22A) or SH3 domain binding function (W525K) have been described [44], [45]. To analyze the role of the different cortactin motifs in *C. burnetii* internalization, we tested two cortactin mutants: W22A (20DDW22 motif mutated to 20DDA22), an NTA mutant that has lost its ability to bind and activate Arp2/3 [29]; and W525K, an SH3 mutant that is unable to bind certain targets such as N-WASP [30]. HeLa cells were transfected with plasmids encoding GFP-cortactin WT, GFP-cortactin W22A or GFP-cortactin W525K, infected with *C. burnetii* at 37°C for 4 h, processed for indirect immunofluorescence and analyzed by fluorescence microscopy (see Methods). We decided to allow 4 h for *C. burnetii* internalization in order to detect the intracellular bacteria with sufficient resolution.

To quantify *C. burnetii* internalization by immunofluorescence we used the conventional double cycle antibody staining protocol for discriminate between extra- and intracellular bacteria (see Methods). In the Fig. 1A (panels d, h, l and p), extracellular bacteria present double staining (green and red pseudocolors) while intracellular ones present single staining (red pseudocolor). The intracellular bacteria are expressed as a percentage of the total number of bacteria per cell (Fig 1B and 1A, panels c, g, k and o). As shown in Fig. 1B, in cells expressing GFP-cortactin W525K, *C. burnetii* internalization was significantly lower than that observed in cells expressing GFP alone. In contrast, expression of the W22A mutant did not affect *C. burnetii* internalization. These results suggest that the SH3 domain of cortactin is critical for *C. burnetii* entry into HeLa cells.

**Figure 4:**
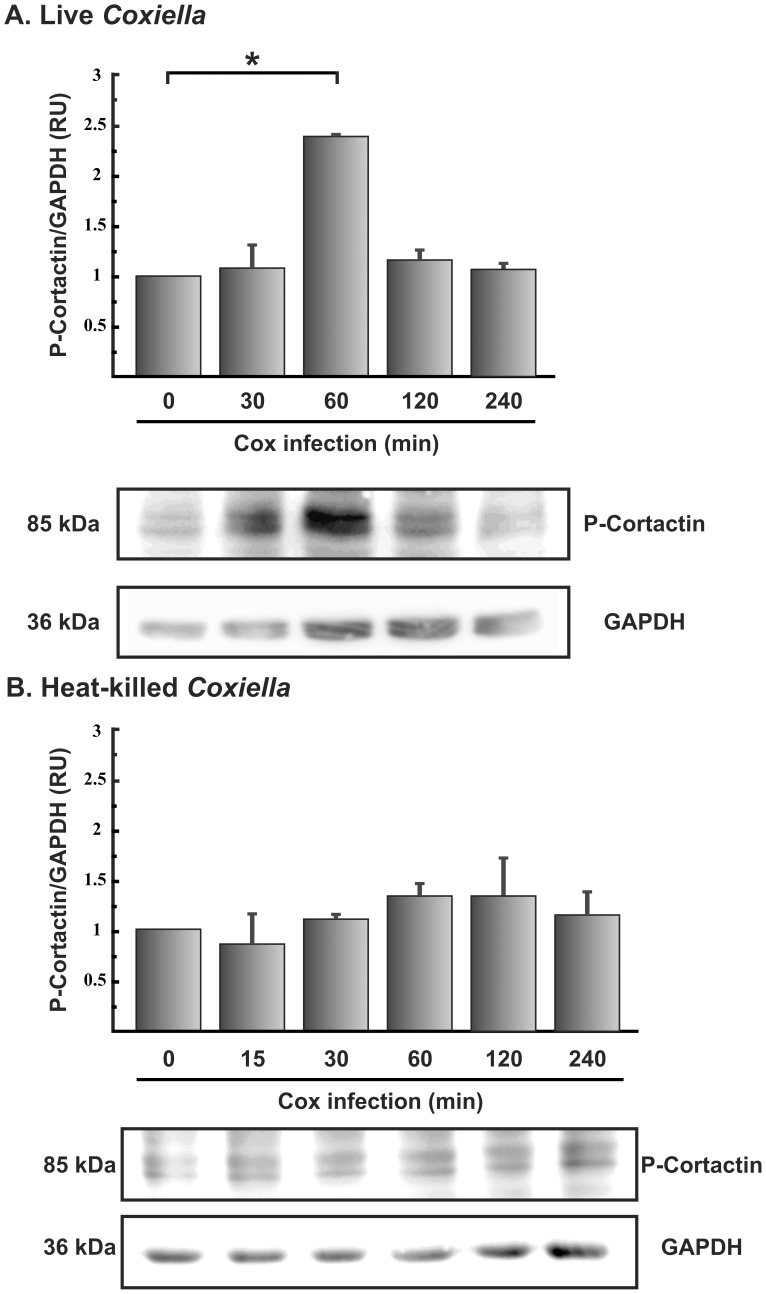
Tyrosine phosphorylation of cortactin during *C. burnetii* infection. Lysates of HeLa cells infected for different lengths of time with live (A) or heat-killed (B) *C. burnetii* were analyzed by SDS-PAGE and Western blot using an antibody against phosphoTyr421-cortactin (P-cortactin) or an anti-GAPDH antibody. 0 min: control HeLa cells incubated in the absence of *C. burnetii*. The data were analyzed with ImageJ software. The ratio between phosphorylated cortactin and GAPDH levels are shown. Results are expressed as means ± SE from at least three independent experiments. *, P<0.05. (RU), relative units.

### A Non-tyrosinephosphorylatable Cortactin Mutant Stimulates *C. burnetii* Internalization

Post-translational modifications such as phosphorylation at tyrosine and serine residues at PST region of cortactin regulate its cellular function. Src kinases and other Tyr kinases phosphorylate human cortactin predominantly at three sites *in vitro*, Tyr421, Tyr470 and Tyr486 (corresponding to Tyr421, Tyr466 and Tyr482 in murine cortactin), while ERK and PAK phosphorylate Ser405/Ser418 and Ser113, respectively [23], [26], [30], [46]–[48]. In addition to that, c-Met and Fer kinases can phosphorylate cortactin on tyrosine residues [47], [49]. The combined mutation of Tyr421, Tyr466, and Tyr482 abolishes tyrosine phosphorylation of cortactin in cells under various conditions [46], [49]. Thus, these Src phosphorylation sites have been the focus of functional characterization. At the same time, several mass spectrometry-phosphoproteomic studies have identified additional phosphorylated tyrosine residues [50], [51]. A number of individual phosphotyrosine sites have been reported independently in different cell types and in response to diverse stimuli, but their regulation and function remain to be investigated. A validated tool to study the role of phosphorylation is to use non-phosphorylatable and phospho-mimetic mutants. To determine whether tyrosine phosphorylation plays a role in internalization, HeLa cells were transfected with a plasmid encoding GFP-cortactin 3F (Y421,466,482F), which encodes a cortactin that cannot be phosphorylated by Src on tyrosines. Transfected HeLa cells were infected with *C. burnetii* at 37°C for 4 h, processed for indirect immunofluorescence and analyzed by fluorescence microscopy. As shown in Fig. 2A, the levels of *C. burnetii* internalization were similar in cells expressing either GFP (control) or GFP-cortactin WT, but significantly higher in cells expressing GFP-cortactin 3F. In contrast, when HeLa cells were transfected with a plasmid encoding pGFP-cortactin 3D (Y421,466,482D), which mimics cortactin phosphorylated by Src, internalization of *C. burnetii* was similar to that in cells expressing GFP-cortactin WT (Fig. 2B). These results suggest that *C. burnetii* internalization is favored when cortactin is dephosphorylated.

**Figure 5:**
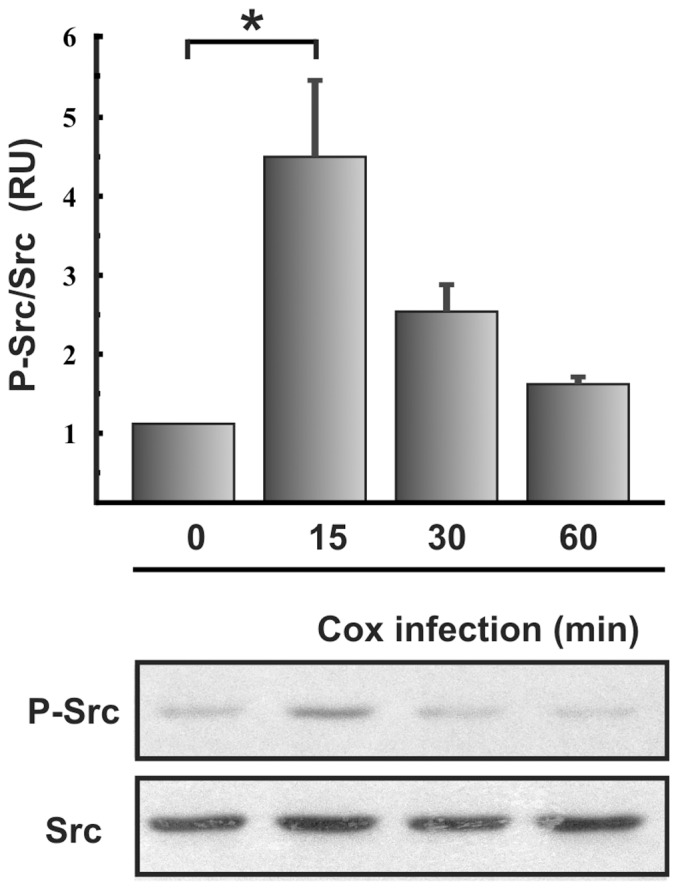
Tyrosine phosphorylation of Src kinase during the *C. burnetii* infection. Lysates of HeLa cells infected with *C. burnetii* for different lengths of time were analyzed by SDS-PAGE and Western blot using anti-phospho-Src (P-Src) and anti-Src antibodies. 0 min: control HeLa cells incubated in the absence of *C. burnetii*. Data were analyzed with ImageJ software. The ratio between phosphorylated and total Src levels is shown. Results are expressed as means ± SE of at least three independent experiments. *, P<0.05. (RU), relative units.

### A Non-serinephosphorylatable Cortactin Mutant Inhibits *C. burnetii* Internalization

Since the cellular function of cortactin is also regulated by phosphorylation of its serine residues, we studied whether serine phosphorylation is important for *C. burnetii* internalization. HeLa cells were transfected with pGFP, pGFP-cortactin WT, pGFP-cortactin 2A or pGFP-cortactin SD. Cortactin 2A (S405,418A) cannot be phosphorylated by ERK, while cortactin SD (S405,418D) mimics the effects of ERK phosphorylation. As shown in Fig. 2C, expression of the ERK-phosphorylation mimic of cortactin led to internalization similar to that in cells expressing GFP or cortactin WT. Interestingly, the cortactin mutant not phosphorylatable by ERK inhibited internalization, indicating that cortactin phosphorylation on serine residues is important for internalization. These results suggest that serine phosphorylation of cortactin regulates internalization of *C. burnetii* during infection.

**Figure 6:**
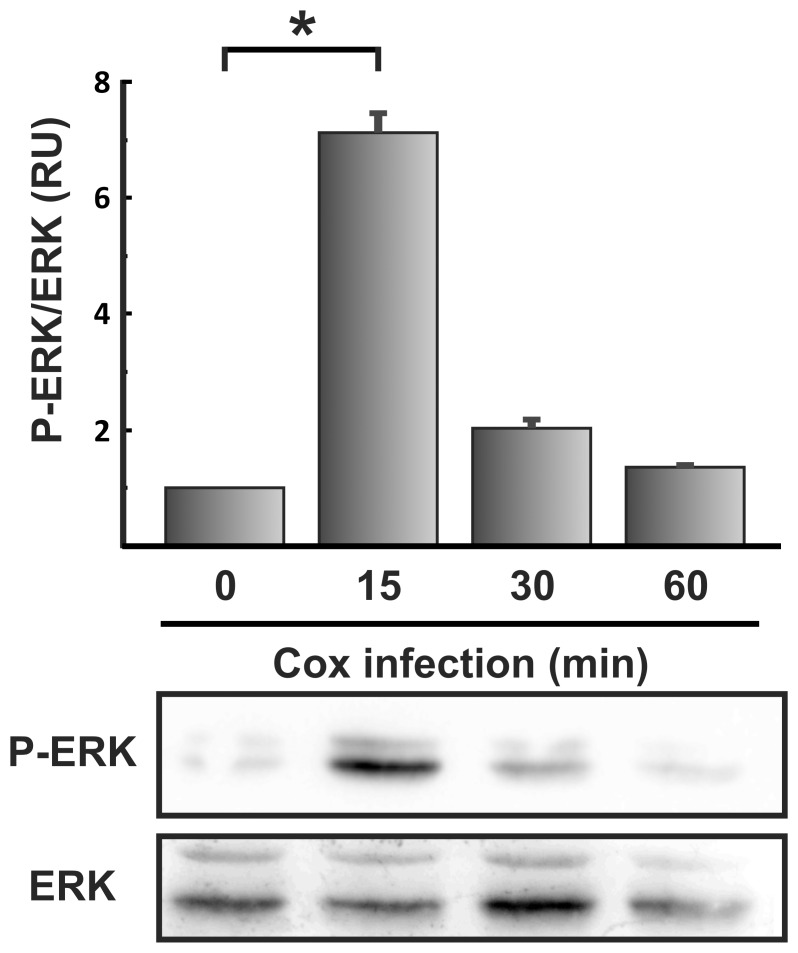
Tyrosine phosphorylation of ERK kinase during *C. burnetii* infection. Lysates of HeLa cells infected with *C. burnetii* for different lengths of time were analyzed by SDS-PAGE and Western blot using anti-phospho-ERK (P-ERK) and anti-ERK antibodies. 0 min: control HeLa cells incubated in the absence of *C. burnetii*. Data were analyzed with ImageJ software. The ratio between phosphorylated and total ERK levels is shown. Results are expressed as means ± SE of at least two independent experiments. *, P<0.05. (RU), relative units.

### Src and ERK Kinases are Involved in *C. burnetii* Internalization

Given that dynamic phosphorylation of tyrosine and serine residues in cortactin is involved in *C. burnetii* uptake and that cortactin is a substrate of kinases of the Src and ERK families, we analyzed directly whether these kinases are involved in *C. burnetii* internalization. HeLa cells were treated during infection with Src kinase inhibitor SU6656 or with PD98059, an inhibitor of MEK kinases which are upstream activators of ERK kinases [52], [53]. Fig. 3A shows extracellular bacteria (panels a, c and e) and total bacteria (panels b, d and f) in untreated or inhibitor-treated cells. Quantification of internalized bacteria is shown in Fig. 3B. Both inhibitors significantly blocked internalization. Similar inhibition of the *C. burnetii* uptake was observed during infection of a macrophage cell line treated with the MEK kinase inhibitor (Fig. S1A and B). These results suggest that kinases of the Src and ERK families are involved in *C. burnetii* internalization.

### *C. burnetii* Induces Phosphorylation of Cortactin on Tyr421 During Infection

Several pathogens have been found to modify the phosphorylation status of cortactin during their interaction with host cells [28]. To determine whether *C. burnetii* causes cortactin phosphorylation during infection, we examined the phosphorylation status of Tyr421 after different infection periods. HeLa cells were infected with *C. burnetii* for different periods of time, and clarified lysates were analyzed by SDS-PAGE and Western blotting using an antibody that specifically recognizes cortactin phosphorylated on Tyr421. Fig. 4A shows that the maximal level of phospho-Tyr421 cortactin was observed at 1 h of infection with live bacteria. Similar cortactin phosphorylation levels were observed during infection of a phagocytic cell (Fig. S2B). In contrast, the level of this form of phosphorylated cortactin did not change significantly when the infection was carried out with heat-killed *C. burnetii* (Fig. 4B). These results suggest that *C. burnetii* induces phosphorylation of cortactin on Tyr421 early during infection.

### Src and ERK Kinases are Activated During *C. burnetii* Infection

Considering that cortactin is phosphorylated on tyrosine residues and that it is a substrate of Src kinase, we reasoned that this enzyme may be activated during *C. burnetii* infection. To investigate this possibility, clarified lysates of HeLa cells infected with *C. burnetii* were analyzed by SDS-PAGE and Western blotting using an antibody that specifically recognizes phospho-Src (pTyr416), the activated form of the kinase. Fig. 5 shows the levels of activated Src and total Src. Src was activated early during infection, within 15 min, after which the level of activated enzyme decreased to basal levels. This result suggests that Src kinase is activated early during *C. burnetii*-host cell interaction.

Cortactin can also be regulated by phosphorylation of serine residues and ERK is one kinase involved in this reaction. To investigate whether ERK is activated during *C. burnetii* infection, lysates of infected HeLa cells were analyzed by SDS-PAGE and Western blotting using an antibody that specifically recognizes phosphorylated ERK. As shown in Fig. 6, ERK was activated at 15 min of infection. Similarly, the phosphorylation in ERK was observed during infection of a phagocytic cell (Fig. S2A). This result suggests that ERK, similar to Src kinase, is activated early during *C. burnetii*-host cell interaction.

## Discussion

Bacterial pathogens manipulate the host cell cytoskeleton to avoid phagocytosis, to invade and/or to become mobile in the host cell cytoplasm. They often interact with actin filaments by modulating the activity of different actin-interacting effectors in the host. One such effector is cortactin, an actin-binding protein that plays a crucial role in the regulation of actin dynamics. Cortactin has been implicated in the infection process of several microbial pathogens [28].

The present study contributes to understanding the role of cortactin in bacterial pathogenesis. We provide evidence that the SH3 domain and serine phosphorylation of cortactin are involved in signal transduction pathways that support the internalization of avirulent *C. burnetti* into non-phagocytic cells, whereas tyrosine phosphorylation of cortactin suppresses this internalization. We also show that Src and ERK kinases are activated during the initial stages of *C. burnetti* infection.

Cortactin stimulates actin polymerization by binding, via its N-terminal domain, the Arp2/3 complex, or by binding, through its C-terminal SH3 domain, N-WASP, a well-known activator of Arp2/3 [28], [30]. We report here that expression in HeLa cells of the cortactin mutant W525K, which carries a mutation in the C-terminal SH3 domain, significantly inhibited *C. burnetii* internalization (Fig. 1), suggesting an important role for this domain in bacterial entry. We hypothesize that avirulent *C. burnetii* requires minor modification of the actin cytoskeleton at the plasma membrane to be internalized, so we propose that SH3 domain-mediated recruitment of N-WASP (and then Arp2/3) is sufficient to stimulate actin assembly and bacterial uptake. Our results are similar to those observed in the formation of the pedestal-like structure during *E. coli* infection. Indeed, expression of the cortactin mutant W525K significantly reduced the number of pedestals induced by enteropathogenic *Escherichia coli* (EPEC) or enterohemorrhagic *E. coli* (EHEC) in HeLa cells [27], [54]. In addition, cell motility can be regulated by cortactin through its C-terminal SH3 domain independently of the presence of the N-terminal portion which is consistent with the ability of the SH3 domain on its own to stimulate N-WASP and actin polymerization *in vitro* [30]. Cortactin cleavage by calpain has also been shown to be important for cell migration [55]. Cells expressing a calpain-resistant cortactin showed reduced migration and increased membrane protrusion. This phenotype was reverted by expression of a calpain-resistant cortactin with the W525K mutation, which suggests that the SH3 domain of cortactin is required for the stimulation of membrane protrusions.

N-WASP activation depends on the phosphorylation status of serine and tyrosine residues in the C-terminal domain of cortactin [30]. Expression of a non-serine-phosphorylatable cortactin mutant impairs pedestal formation in cells infected with EPEC or EHEC [54]. These results suggest that ERK phosphorylation of cortactin contributes to pedestal formation. In HeLa cells expressing the same cortactin mutant we observed significant inhibition of bacterial uptake (Fig. 2). Thus, similarly to pedestal formation induced by *E. coli*, entry of *C. burnetii* into HeLa cells requires serine phosphorylation of cortactin. Our conclusion is also supported by the observations that ERK1/2 kinases were transiently activated early during HeLa cell infection and then later deactivated (Fig. 6), and that HeLa cell treated with the inhibitor of MEK-dependent ERK1/2 activation showed a reduction in *C. burnetii* internalization (Fig. 3).

In our experimental model, expression of a non-tyrosine-phosphorylatable cortactin mutant increased *C. burnetii* internalization (Fig. 2). However, pedestal formation induced by EPEC was reduced in HeLa cells expressing the same mutant [54], [56]. Moreover, we observed cortactin phosphorylation on Tyr421 at 1 h of infection with *C. burnetii*, after which the phosphorylation returned to basal levels (Fig. 4). Similar kinetics of tyrosine phosphorylation-dephosphorylation were observed in HeLa cells infected with pre-activated EHEC [57]. In vitro experiments have shown that cortactin phosphorylated by Src does not interact with and activate N-WASP, which leads to inhibition of pedestal formation [30], [57]. Based on these results, we can speculate that during *C. burnetii* infection of HeLa cells, cortactin must be dephosphorylated on its tyrosine residues in order to interact with N-WASP, leading to actin remodeling and bacterial internalization. At the same time, tyrosine dephosphorylation of cortactin increases its actin-crosslinking activity in vitro [58]. Therefore, cortactin dephosphorylated on its tyrosine residues may cross-link small actin filaments, forming a discrete actin meshwork close to the bacterial attachment site, which then allows *C. burnetii* internalization.

Cortactin can be tyrosine-phosphorylated not only by Src kinases but also by Abl kinases [59]. Abl kinases can be activated by autophosphorylation and by phosphorylation by Src family kinases [60], [61]. We observed that Src kinase was activated at 15 min of infection and then the levels of activated enzyme decreased to basal levels (Fig. 5), while cortactin was phosphorylated on Tyr421 at 1 h of infection. We also observed that the pharmacological Src inhibitor decreased *C. burnetii* internalization (Fig. 3). While these observations may be due to the direct action of Src kinase on cortactin, they may also be due to the action of Abl kinase, activated by Src. Internalization of *Chlamydia trachomatis* also involves cortactin, and this protein is phosphorylated at 1 h of infection by Abl kinases but not Src [62]. *Shigella* entry into host cells also requires activation of Abl kinases [63]. Therefore we speculate that during *C. burnetii* infection, Abl kinases are activated to phosphorylate cortactin. On the other hand, the strong effect of the chemical inhibition of Src kinase could indicate that other Src substrates apart from cortactin might participate in *C. burnetii* entry.

We show here that during *C. burnetii* infection, Src is transiently activated and then inactivated, and cortactin is tyrosine-phosphorylated and then dephosphorylated. We detected that *C. burnetii* entry induces the tyrosine phosphorylation of cortactin at early time points, with a maximum peak around 60 min (Fig. 4). In addition to that, we found that the 3F cortactin mutant with non-phosphorylatable tyrosines enhances *C. burnetii* entry at 4 h after infection (Fig. 2). This seems to indicate that the tyrosine phosphorylation of cortactin is required at the initial steps while it would inhibit entry at later time points. These processes are similar to those observed during infection of gastric epithelial cells by *Helicobacter pylori*. This pathogen promotes an early but transient phosphorylation of cortactin. The infected cells become scattered and elongated, and this phenotype depends on Src phosphorylation of CagA (a protein secreted by a type IV secretion system), which inactivates c-Src and leads to cortactin dephosphorylation by an unknown mechanism [64]. It is tempting to speculate that *C. burnetii* internalization occurs by a mechanism similar to that of *H. pylori*. To our knowledge, *C. burnetii*, *H. pylori* and EHEC are the only three pathogens known to induce dephosphorylation of cortactin during host cell infection. Recently, *H. pylori* has been shown to induce cortactin phosphorylation on serines in a CagA-independent manner, and this form of cortactin stimulates actin rearrangement and cell elongation [65]. We show here that expression of a cortactin mutant lacking phosphorylatable serines inhibited *C. burnetii* internalization, which suggests that serine phosphorylation of cortactin is necessary for *C. burnetii* entry.

THP-1 monocytes infected with virulent *C. burnetii* exhibit intense membrane protrusions associated with major actin cytoskeleton reorganization, while infection with avirulent bacteria induced a few membrane folds without significantly affecting cell morphology [66]. Although it was not the focus of the present study, we think that the membrane folds stimulated by avirulent *C. burnetii* result from a modest actin cytoskeleton rearrangement that facilitates bacterial uptake. Meconi and collaborators also showed that actin cytoskeleton reorganization is associated with tyrosine phosphorylation of the Src family kinases Hck and Lyn very early during infection of THP-1 cells with virulent, but not avirulent, *C. burnetii* [67]. In the present study, using an anti-phospho-Src antibody that recognizes several members of the Src family, including Hck and Lyn, we found that Src was activated early during infection with avirulent bacteria. Meconi et al. also found, using an anti-phosphoTyr monoclonal Ab, that virulent *C. burnetii,* but not avirulent bacteria, stimulate the tyrosine phosphorylation of several proteins. Using a similar experimental strategy, we observed a significant labeling of proteins with masses around 85 kDa at 1 h of HeLa infection with avirulent *C. burnetii* (data not shown). Cortactin migrates as a doublet of 80 and 85 kDa in SDS-PAGE [68], [69]. We cannot rule out the possibility that the differences between our results and those of Meconi et al. are due to the different cell types used.

In conclusion, our results indicate that serine phosphorylation of cortactin and its SH3 domain are involved in a signal transduction mechanism that favors *C. burnetii* uptake, while tyrosine phosphorylation suppresses this uptake. Our results suggest that a complex series of events occurs during *C. burnetii* internalization into non-phagocytic cells. Early after infection, Src and ERK kinases may phosphorylate unknown substrates, perhaps other kinases such as Abl that in turn phosphorylate tyrosine residues in cortactin. This may regulate an early step in internalization. At a later stage, tyrosine dephosphorylation and serine phosphorylation of cortactin take place, which regulates a later step of internalization. In this way, tyrosine phosphatases and serine kinases from the host cell and/or *C. burnetti* regulate the phosphorylation status of cortactin to favor *C. burnetti* entry into the host cell. Thus, the results reported here indicate that dynamic phosphorylation of cortactin is important for *C. burnetii* internalization during infection.

## Supporting Information

Figure S1 **ERK kinase is involved in** ***C. burnetii*** **internalization.** (A) RAW macrophages were incubated for 1 h at room temperature with 0.05% DMSO (control) or 15 µM PD 98059 (MEK-ERK inhibitor). Then the cells were infected for 2 h with *C. burnetii* in the presence of the inhibitor. Cells were fixed and processed for indirect immunofluorescence using a specific anti-*C. burnetii* antibody (see Methods). Bars, 10 µm. (B) Quantification of *C. burnetii* internalized by treated RAW macrophages. Results are expressed as means ± SE of three independent experiments. ***, P<0.01. (%), percentage of the total number of bacteria.
(TIF)

Figure S2 **Tyrosine phosphorylation of cortactin and ERK proteins during** ***C. burnetii*** **infection.** Lysates of RAW macrophages infected with *C. burnetii* for different lengths of time were analyzed by SDS-PAGE and Western blot using antibodies against phosphoTyr421-cortactin (P-cortactin), phospho-ERK (P-ERK) or GAPDH. 0 min: control RAW macrophages incubated in the absence of *C. burnetii*. Data were analyzed with ImageJ software. The ratio between phosphorylated ERK and GAPDH (A), and phosphorylated cortactin and GAPDH (B) levels are shown. The results are representative of two independent experiments. (RU), relative units.
(TIF)
