# Supplementary figures and images for: Cortactin Is Involved in the Entry of Coxiella burnetii into Non-Phagocytic Cells
Source: PLoS One. 2012 Jun 22;7(6):e39348. doi: 10.1371/journal.pone.0039348 (PMC3382237; doi:10.1371/journal.pone.0039348)

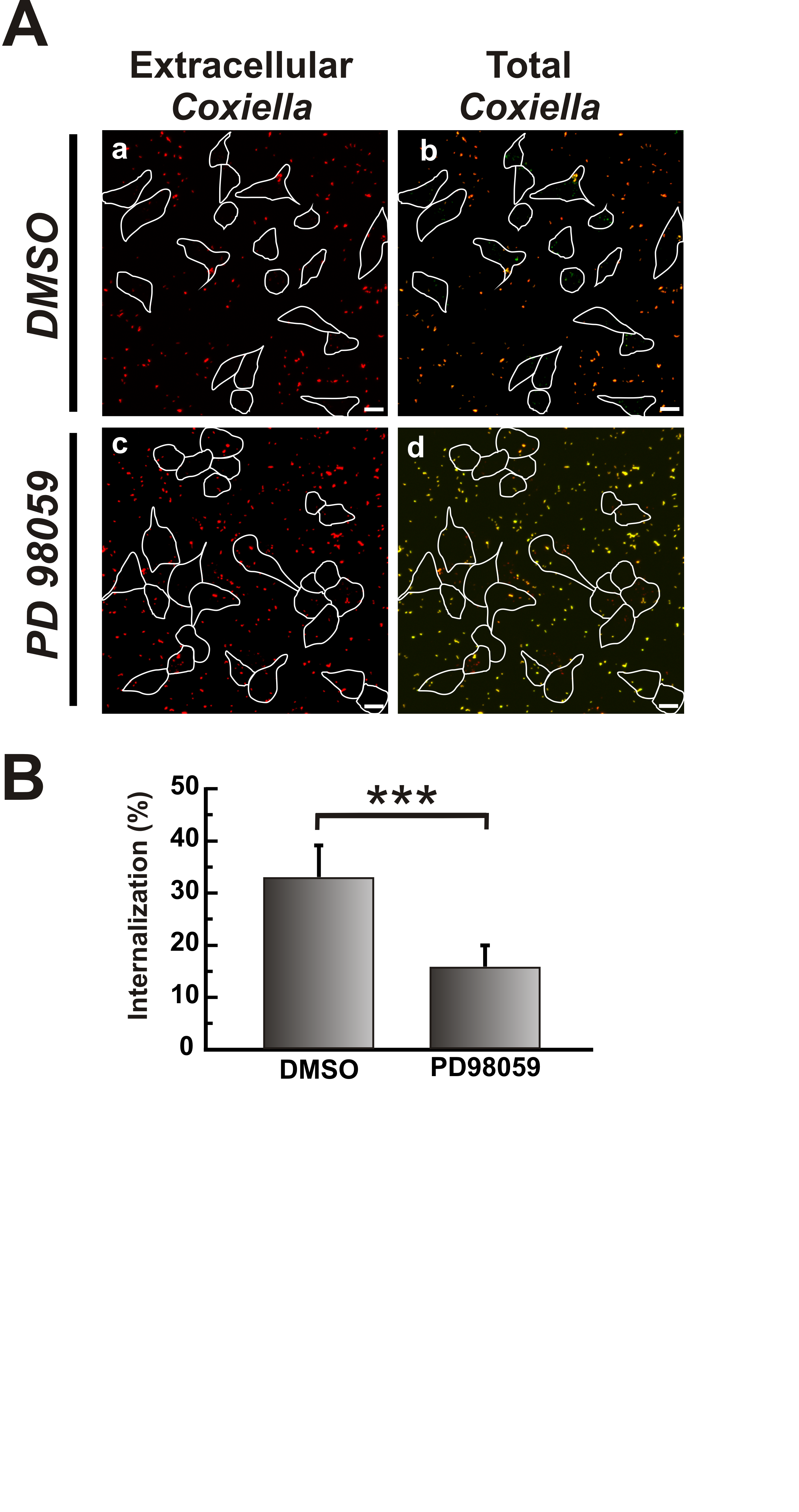

Supplement: Figure S1 — ERK kinase is involved in C. burnetii internalization. (A) RAW macrophages were incubated for 1 h at room temperature with 0.05% DMSO (control) or 15 µM PD 98059 (MEK-ERK inhibitor). Then the cells were infected for 2 h with C. burnetii in the presence of the inhibitor. Cells were fixed and processed for indirect immunofluorescence using a specific anti-C. burnetii antibody (see Methods). Bars, 10 µm. (B) Quantification of C. burnetii internalized by treated RAW macrophages. Results are expressed as means ± SE of three independent experiments. ***, P<0.01. (%), percentage of the total number of bacteria. (TIF) [file pone.0039348.s001.tif]

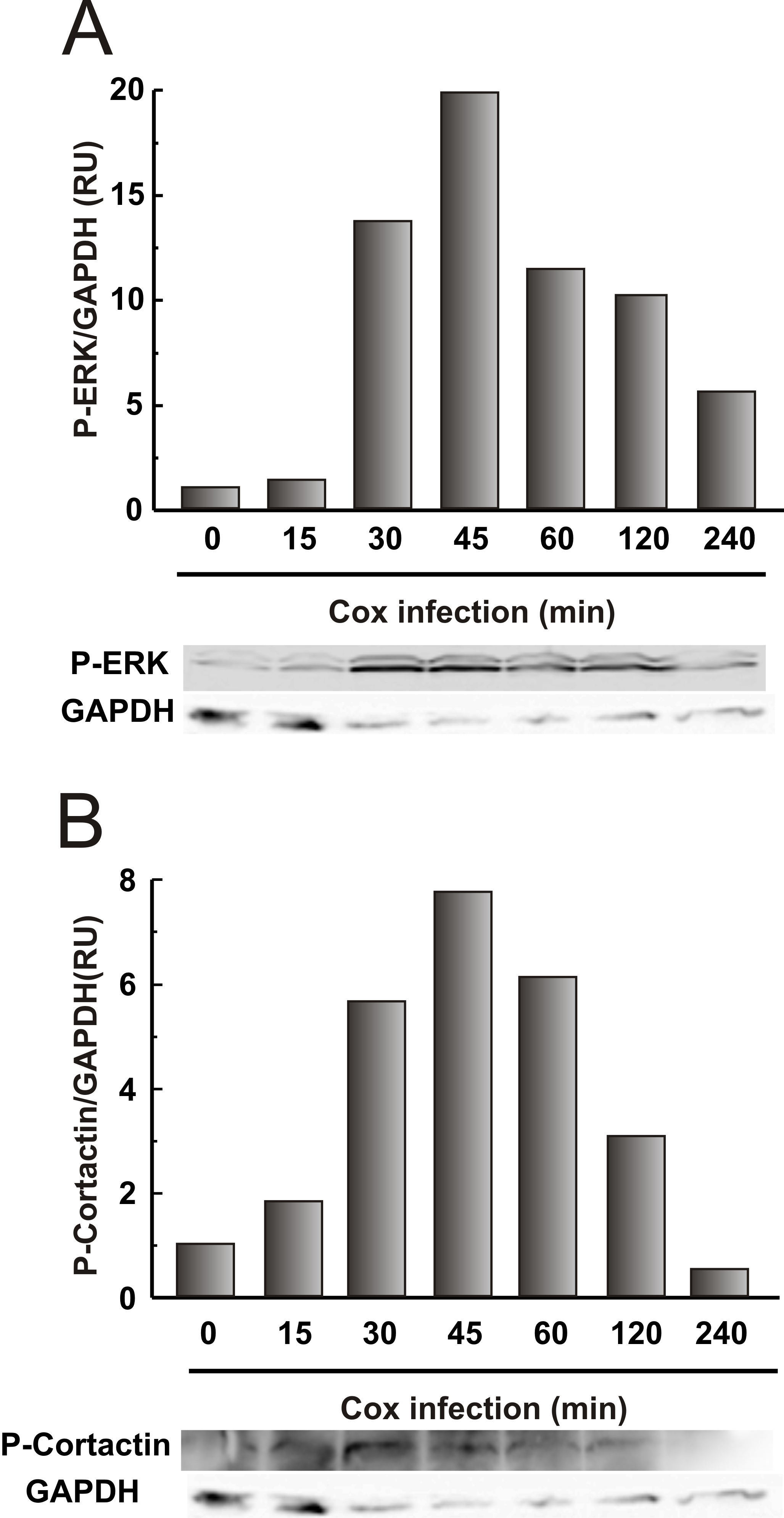

Supplement: Figure S2 — Tyrosine phosphorylation of cortactin and ERK proteins during C. burnetii infection. Lysates of RAW macrophages infected with C. burnetii for different lengths of time were analyzed by SDS-PAGE and Western blot using antibodies against phosphoTyr421-cortactin (P-cortactin), phospho-ERK (P-ERK) or GAPDH. 0 min: control RAW macrophages incubated in the absence of C. burnetii. Data were analyzed with ImageJ software. The ratio between phosphorylated ERK and GAPDH (A), and phosphorylated cortactin and GAPDH (B) levels are shown. The results are representative of two independent experiments. (RU), relative units. (TIF) [file pone.0039348.s002.tif]
